# Osteoblast inhibition by chemokine cytokine ligand3 in myeloma-induced bone disease

**DOI:** 10.1186/s12935-014-0132-6

**Published:** 2014-12-12

**Authors:** Rong Fu, Hui Liu, Sijie Zhao, Yihao Wang, Lijuan Li, Shan Gao, Erbao Ruan, Guojin Wang, Huaquan Wang, Jia Song, Zonghong Shao

**Affiliations:** Department of Hematology, Tianjin Medical University General Hospital, 154 Anshao Street, Heping District, Tianjin 300052 PR China

**Keywords:** Chemokine cytokine ligand 3, Myeloma bone disease, Osteoblast, Runx2, Osterix

## Abstract

**Background:**

Multiple myeloma is a hematologic malignancy characterized by the accumulation of monoclonal plasma cells in the bone marrow. A common manifestation of the disease is myeloma bone disease (MBD), which is caused by increased osteoclastic bone resorption and decreased bone formation. The chemokine cytokine ligand 3 (CCL3) is a pro-inflammatory protein and chemokine that stimulates osteoclasts in MBD. However, little is known about the effect of CCL3 on osteoblasts (OB).

**Methods:**

The OBs are induced from patients with MBD and healthy donors, cultured in vitro, and identified by histochemistry. The effects of CCL3 and CCL3 antibody on the OBs in vitro are observed. The CCL3 receptor (CCR1), osteocalcin (OCN), runt-related transcription factor 2 (Runx2), and osterix (Osx) are detected using flow cytometry, enzyme-linked immunosorbent assay, and real-time PCR.

**Results:**

Proliferation and osteogenic potential of the OB in patients with MBD are suppressed. Moreover, the CCR1 expression is significantly higher in patients with MBD than in normal controls. The OCN level, quantity of calcium nodules, and Runx2 and Osx levels decrease after CCL3 stimulation, which indicates that CCL3 inhibits OB function. Furthermore, CCL3 antibody partially restores OB activity through the upregulation of the OCN, Runx2, and Osx.

**Conclusions:**

CCL3 contributes to the OB/OC imbalance by inhibiting OB differentiation and function in MBD.

## Background

Majority of patients with multiple myeloma (MM) suffer from bone osteolytic lesions also called myeloma bone disease (MBD). MBD may lead to severe complications including pain and fracture. The formation of new bones, which normally occurs in sites of previous bone destruction, is absent or markedly suppressed [1]. Consequently, the osteolytic lesions rarely heal because of the MM-induced suppression of osteoblast (OB) activity even when the patients have prolonged remission and the MM cells are not detectable. The mechanism of OB suppression remains unclear until now.

The bone marrow stromal cells, OBs, osteoclasts (OC), endothelial cells and immune cells in a normal condition regulate each other’s function through direct cell-to-cell contact, cytokine secretion, or extracellular matrix protein deposition. Uncoupled bone remodeling results from the imbalanced activity of OCs and OBs in patients with MM. The chemokine cytokine ligand 3 (CCL3), which is also known as the macrophage inflammatory protein 1-alpha (MIP-1α), is one of the factors responsible for decreased bone formation. CCL3 is a low molecular weight monokine with inflammatory and chemokinetic properties characterized as an osteoclast stimulatory factor in MM [2,3]. CCL3 is elevated in the bone marrow plasma cells of patients with active MM and correlated with the presence of osteolytic lesions [2]. Serum MIP-1α correlates with survival and bone resorption markers, which suggests that MIP-1α possibly contributes to MBD pathogenesis and tumor growth as reflected by its effect on survival [4,5]. MIP-1α also stimulates proliferation, migration, and survival of plasma cells [6,7]. hMIP-1α receptors (i.e., CCR1 and CCR5) are expressed in human bone marrow cells [8]. However, no data are presently available on the effects of CCL3 on OB. Thus, this study investigates the effect of CCL3 on the OB of patients with MBD.

## Methods

### Study subjects

A total of 21 newly diagnosed patients with MBD (i.e., 13 males and 8 females) were enrolled. The participants were selected as inpatients in the Hematology Department of the General Hospital of the Tianjin Medical University between January and December 2013 according to the International Myeloma Workgroup criteria. The patient median age was 61 years old (range: 44–74 years). Eight healthy volunteers with a median age of 40.5 years old (range: 19–65 years) were included as normal controls. Bone marrow aspirations were collected from all the patients diagnosed with MBD and from normal controls. This study was approved by the Ethics Committee of the Tianjin Medical University. Written informed consent was obtained from the patient for the publication of this report and any accompanying images.

### Cell culture

The bone marrow mononuclear cells (BMMNC) were separated using Ficoll–Hypaque density sedimentation. The BMMNCs were cultured in Dulbecco’s modified Eagle/F12 medium supplemented with 15% fetal bovine serum (Gibco, Darmstadt, Germany), 1 × 10^−7^ mol/L dexamethasone, 0.05 g/L vitamin C, 0.01 mol/L β-sodium glycerophosphate, 100 U/mL penicillin (Gibco), and 100 ug/mL streptomycin (Gibco). Non-adherent cells were removed the next day, and the media were replaced every other day. Adherent BMMNCs were cultured at 37°C in an atmosphere containing 5% CO_2_. The OBs were counted and seeded in 24-well plates at a plating density of 1 × 10^4^ cells/cm^2^. Trypsin was used to detach three wells of OB for cell count every second day. The number of cells was used to draw the OB growth curve. The OB doubling time (DT) was calculated using the following formula:

$$ DT=t\times \left[1\mathrm{g}2/\left(1\mathrm{g}Nt-1\mathrm{g} No\right)\right] $$

where *t* is the culturing period (h); *No* is the cell density when the cells were seeded; and *Nt* is the cell density when the cells were cultured after *t* hours.

The OBs were divided into three groups: group I (i.e., blank group), group II (i.e., the OBs were cultured with CCL3; 50 ng/L), and group III [i.e., the OBs were cultured with CCL3; 50 ng/L; and a neutralizing antibody against CCL3 (R&D Systems) with 5 μg/L concentration]. The changes in the OB osteogenic potential and biological characteristics were observed after intervention.

### ALP activity and mineralization assays

The ALP expression was an early osteoblast marker detected using the ALP Staining Kit (Sigma Aldrich, Taufkirchen, Germany). The expression was utilized to confirm the presence of OB. Von Kossa staining was performed to confirm that the OB synthesized and mineralized an extracellular matrix. The mineralized nodules were found in both groups cultured for three weeks. The amount of mineralized nodules in each patient was counted and used as an indicator of OB function.

### Flow cytometry

The OBs were suspended in Dulbecco’s phosphate-buffered saline (PBS) and incubated with antibodies against mouse IgG_1_-PE,mouse IgG_1_-APC, mouse IgG_1_-FITC and mouse IgG_1_-PerCP (BD, Franklin Lakes, NJ, USA) as a negative control. Antibodies against CCR1-PE,CD138-FITC,CD45-APC and CD34-PerCP (BD) were used to stain the experimental sample. After incubation in the dark at 4°C for 30 min,the cells were washed twice with PBS. At least 5 × 10^4^ cells were acquired and analyzed using a FACSCalibur flow cytometer (BD Biosciences). OBs are marked as CD138^−^CD45^−^CD34^−^ cells.

### Enzyme-linked immunosorbent assay

The OBs were cultured for 3 days, and the supernatants were harvested. The OB-secreted osteocalcin (OCN) level in the supernatants was assessed using the N-MID OCN ELISA Kit (R&D Systems, Inc., Minneapolis, MN, USA). Diluted standards and patient serum (100 μl) were added in duplicate and incubated at 37°C for 1 h. Then, the wells were washed 5 times using a microplate washer. Next, HRP was added to each well. After incubation at 37°C for 30 min, the wells were washed 5 times. Then, TMB solution was added to each well, and the samples were incubated in the dark at room temperature for 20 min. Finally, a stop solution was added, and the OD was read at 450 nm within 15 min.

### Quantitative real-time PCR

The total RNA from the OB of each group was extracted using the TRIzol reagent (Invitrogen). The TIANScript RT Kit (TIANGEN, Beijing, China) was utilized to reverse-transcribe 1 μg of RNA. Table 1 presents the primer sequences. These primer sequences were designed and synthesized by Sangon Biotech (Shanghai, China).

**Table 1:** **Primer sequences**

**Target**	**Sense and anti-sense sequences**	**Bp**
Runx2	F: 5′-AACCACAGAACCACAAGTGCG-3′	119 bp
	R: 5′-AAATGACTCGGTTGGTCTCGG-3′	
Wnt3a	F: 5′-GCCCCCACTCGGATACTTCTTACTC-3′	226 bp
	R: 5′-CTCCTGGATGCCAATCTTGATG-3′	
Osterix (SP17)	F: 5′-CTGCGGGACTCAACAACTCT-3′	194 bp
	R: 5′-TGGGAAAAGGGAGGGTAATC-3′	
β-Actin	F: 5′-TTGCCGACAGGATGCAGAA-3′	100 bp
	R: 5′-GCCGATCCACACGGAGTACT-3′	

Bp: base pairs.

The quantitative real-time PCR was performed using the Bio-Rad iQ 5 Real-time System (Bio-Rad, Hercules, CA, USA). The SYBR Green (Invitrogen) was used as a double-strand DNA-specific dye. The amplification utilized 40 cycles at 95°C for 10 s and 58°C for 20 s with the extension at 72°C for 30 s. β-Actin was employed as the housekeeping gene to standardize the targeted mRNA expression. The runt-related transcription factor 2 (Runx2), Wnt, and osterix (Osx) levels were calculated using the 2^−△△Ct^ method [(Ct, target gene Ct, β-actin)_sample_ − (Ct, target gene Ct, β-actin)_control_] after normalizing the data according to the β-actin mRNA expression.

### Statistical analysis

All data were expressed as mean and standard deviation. The SPSS 16.0 software was used to perform all statistical analyses. The differences between the means of the data were calculated using Student’s *t*-test. The differences among the groups were analyzed by employing the one-way analysis of variance followed by Newman–Keuls multiple comparison test. *P* <0.05 was considered statistically significant.

## Results

### Decrease of proliferation and osteogenic potential of OB from MBD

**Figure 1:**
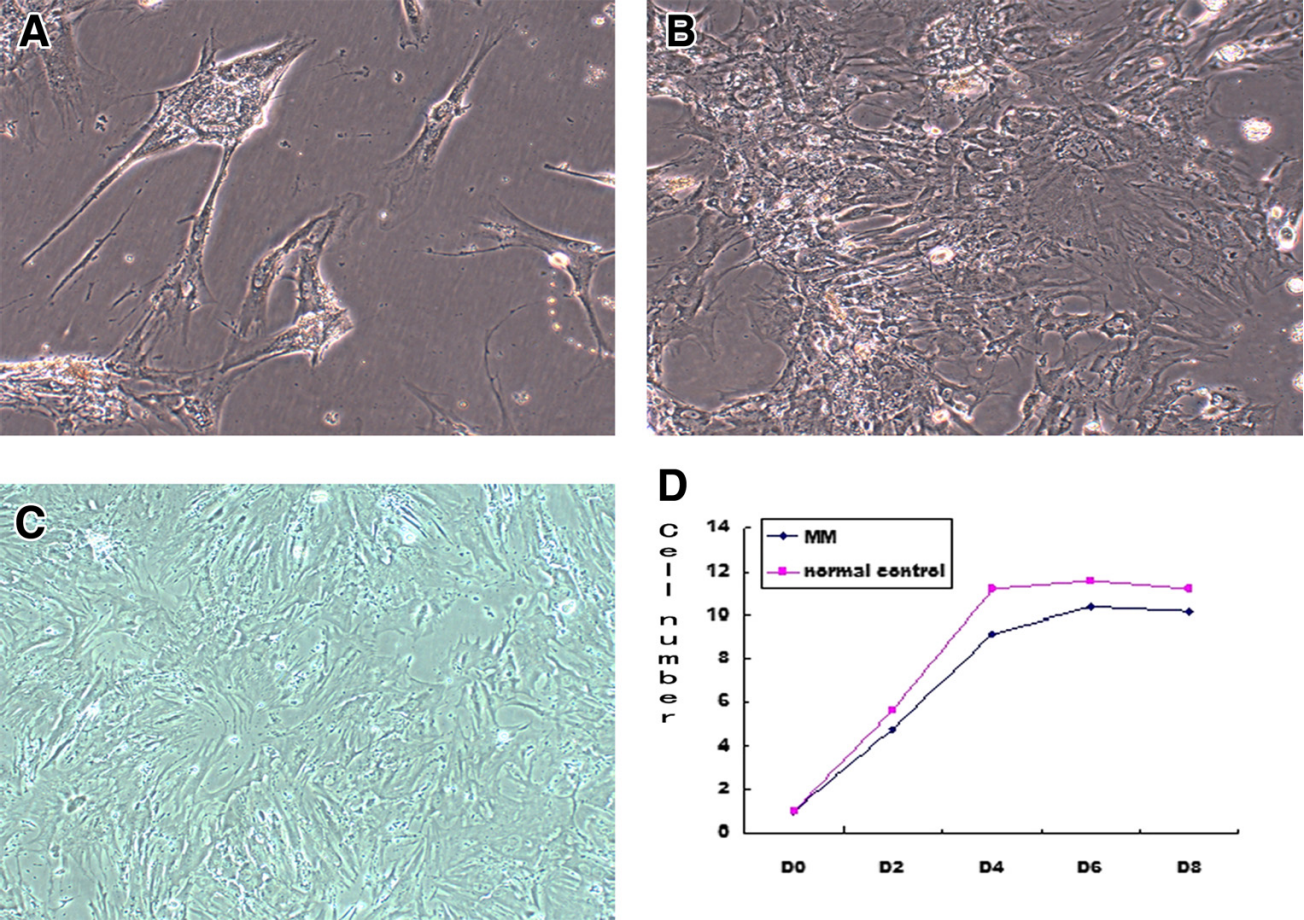
**Morphology of OBs. (A)** The shape of the primary cells nearly resembles that of a spindle and a polygon. **(B)** The primary cultures reach confluency by day 7 and exhibit dense clusters at day 10. **(C)** OB morphology after subculture. All subcultures reach confluency after 4 days to 7 days and form continuous cell multilayers. The latter cultures form isolated or interconnected and multilayered islands of cells widely dispersed on the flask surface. **(D)** The OB growth curve illustrates that the proliferative rate of the OB from patients with MBD are constitutively suppressed.

**Figure 2:**
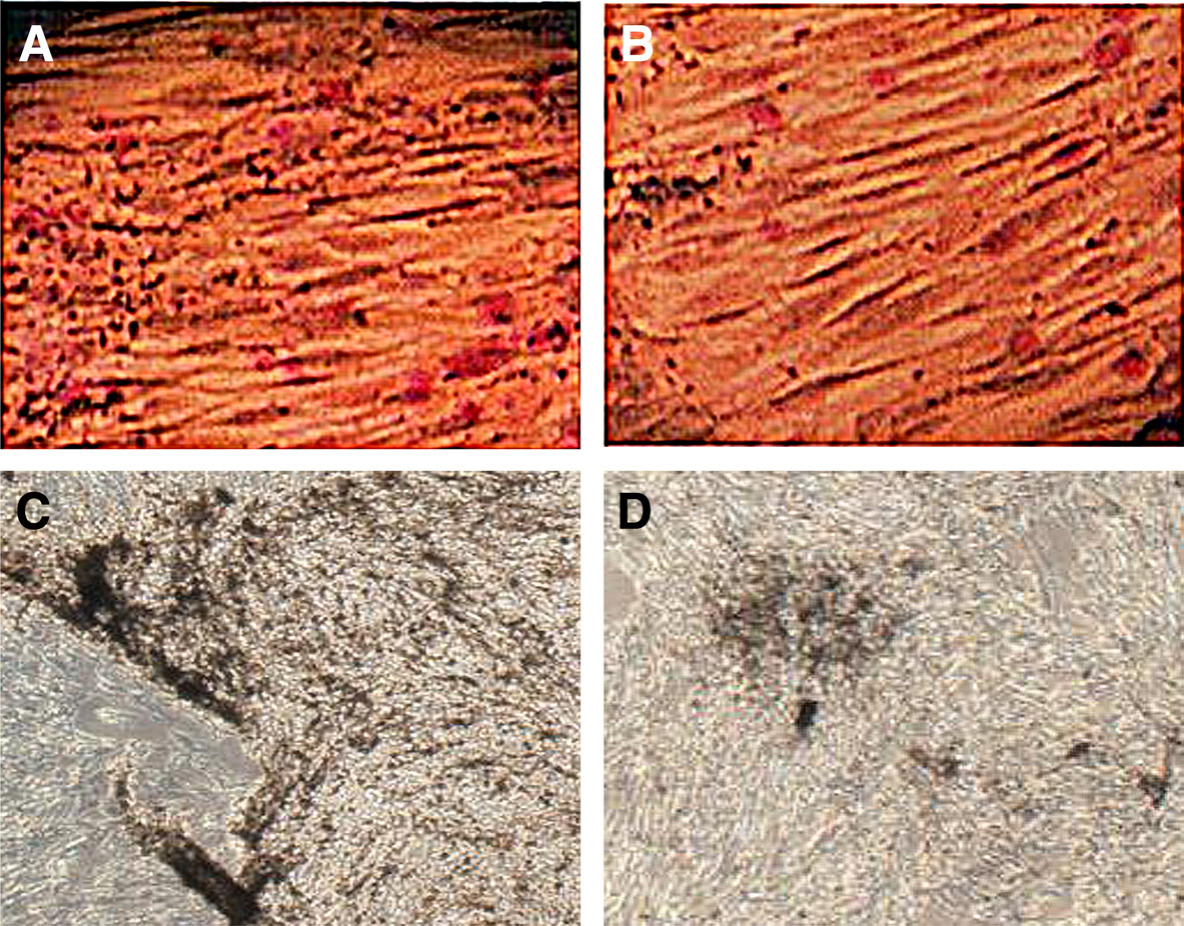
**OBs are identified through (A, B; C, D) ALP and Von Kossa staining.** The number of positive cells of **(B)** the patients with MBD is less than that of **(A)** the normal controls. The mineralized bone-like nodules in **(D)** the patients with MBD are less than those of **(C)** the normal controls.

The OBs from the BM of patients with MBD were cultured in vitro using dexamethasone, β-sodium glycerophosphate, and vitamin C. The shape of the cells nearly resembles that of a spindle and a polygon (Figures 1A–C). The OB growth curve showed that the proliferative rate of the OB from the patients with MBD was constitutively suppressed (Figure 1D). ALP and Von Kossa staining positively identified the OB. The number of positive cells and the amount of mineralized bone-like nodules of the patients with MBD were less than those of the normal controls (Figure 2). The DT was about 22 and 20 h in patients with MBD and normal controls, respectively. Moreover, fewer cells were observed from patients with MBD [(9.1 ± 3.28) × 10^4^/mL] compared with the normal controls [(11.2 ± 2.73) × 10^4^/mL] (*P* <0.05) 4 days after subculture. The proliferative rate of the OB from patients with MM was constitutively suppressed.

### Increase of CCL3 receptor expression on OB in MBD

**Figure 3:**
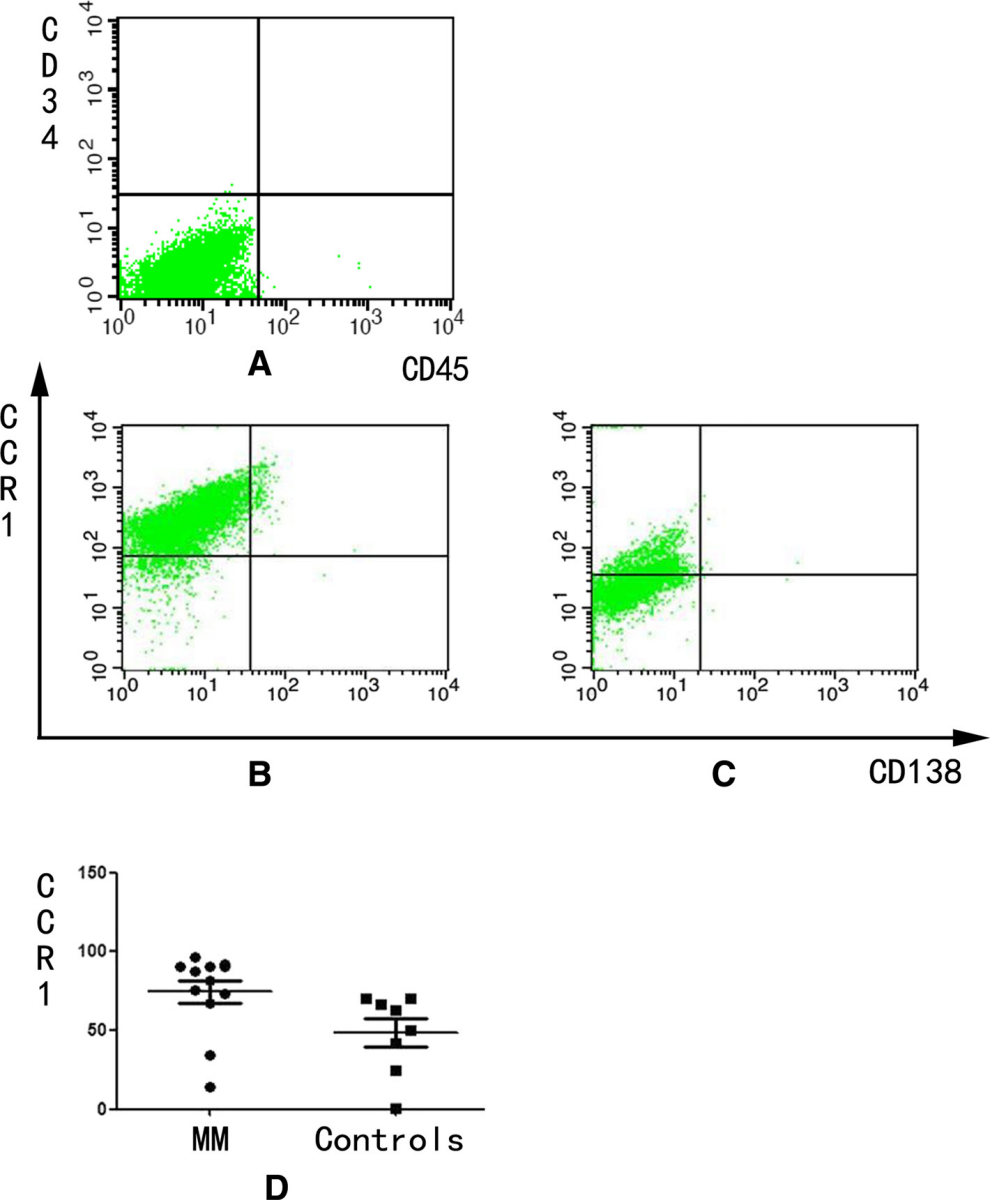
**CCR1 expression is assessed using flow cytometry.** The OB is marked as CD45^−^CD34^−^CD138^−^ **(A)**, which both express CCR1 in **(B)** the patients with MBD and **(C)** normal controls. **(D)** The CCR1 level is significantly higher in patients with MBD (74.48 ± 7.31) % than in the normal controls (48.35 ± 8.81) % (*P* <0.05).

The CCL3 receptor expression (i.e., CCR1) was detected on both OB derived from the patients with MBD and normal controls. The CCR1 expression on OB prompted us to evaluate the effect of CCL3 on the development and activity of OB. The CCR1 level on the OB was significantly higher in patients with MBD (74.48 ± 7.31%) than in the normal controls (48.35 ± 8.81%) (*P* <0.05) (Figure 3). CCL3 played an important role in regulating the OB of patients with MBD.

### CCL3 inhibition of OB function through impaired mineralization and downregulation of the OCN level

**Figure 4:**
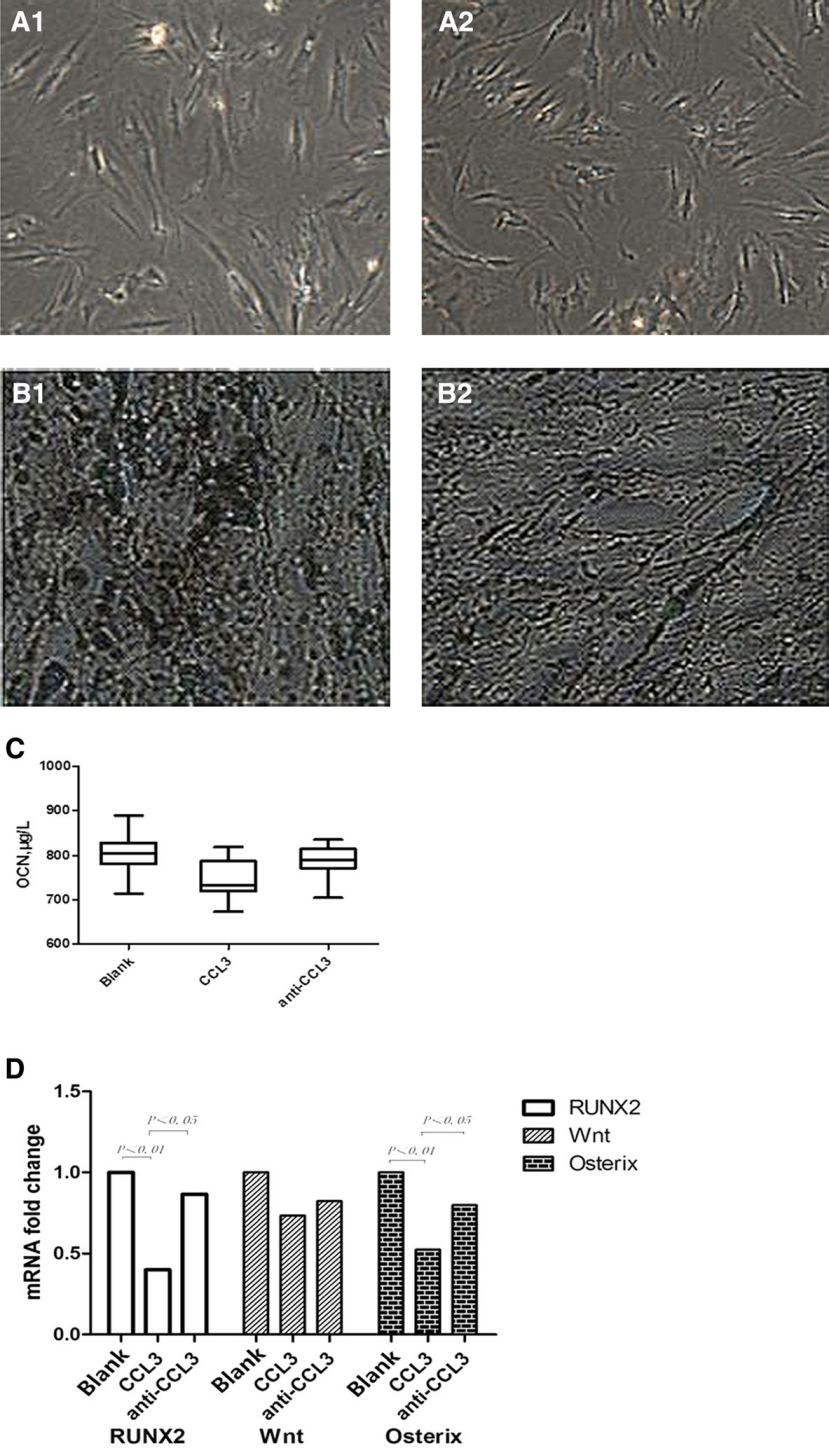
**Effect of CCL3 on the OB of patients with MBD. (A)** The OB morphology is not affected by CCL3. The OB of the blank group (A_1_) is not different from the OB applied with the CCL3 treatment (A_2_). **(B)** The mineralized bone-like nodules of the OB in the CCL3 group (B_2_) are less than that in the blank group (B_1_) (*P* <0.05). **(C)** The OCN level secreted by the OB is detected using ELISA. The secreted OCN reduces after stimulation with CCL3 (*P* <0.05) and is partially restored by CCL3 antibody treatment (*P* <0.05). **(D)** Runx2 and Osx gene expressions in the OB assessed using the quantitative PCR. The expressions are significantly downregulated with CCL3 treatment (*P* <0.01) but promoted after the CCL3 antibody is used (*P* <0.05). The Wnt expression exhibits no change with or without the CCL3 antibody.

The OBs were cultured with or without CCL3 (50 ng/L) for 6 days in vitro. The OB quantity and function were subsequently observed. Matrix formation and mineralization were also observed through histochemistry. The OCN level in the supernatants was detected by ELISA. No change in the OB quantity was found with or without CCL3, whereas the OB function was inhibited with CCL3. The OB quantity co-cultured with CCL3 for 6 days was (2.42 ± 0.22) × 10^5^/mL. This value was not different with that of the blank group (2.82 ± 0.19) × 10^5^/mL (Table 2). The OCN level cultured with CCL3 for 6 days was (750.643 ± 41.116) μg/L, which was significantly lower than that in the blank group [(803.375 ± 40.654) μg/L] (*P* <0.05) (Figure 4). The amount of mineralized nodules (5.42 ± 0.39/HPF) cultured with CCL3 was also significantly lower than in the blank group (9.35 ± 0.62/HPF) (*P* <0.05) (Table 2).

**Table 2:** **Quantity and function of OB cultured with CCL3 and anti-CCL3**

	**Number of cases**	**Number of cells (10** ^**5**^ **/ml)**	**Number of mineralized nodules (/HPF)**	**OCN (μg/L)**
Blank	21	2.82 ± 0.19	9.35 ± 0.62	803.375 ± 40.654
CCL3	21	2.42 ± 0.22	5.42 ± 0.39^*^	750.643 ± 41.116^**^
Anti-CCL3	21	2.49 ± 1.23	6.88 ± 1.75	787.358 ± 32.063^#^

^*^Significantly lower than that in the blank group. **Significantly lower than that in the blank group. ^#^Significantly higher than that in the CCL3 group.

### Runx2 and Osx downregulation in OB of patients with MBD

The mRNA expressions of Runx2, Wnt, and Osx were analyzed to observe OB differentiation. These expressions were critical transcription factors for early and late OB differentiation [9]. The mRNA in the blank group after CCL3 exposure in culture was 1.00, whereas that of Runx2, Wnt, and Osx were 0.4002 ± 0.3734, 0.7341 ± 0.4432, and 0.5242 ± 0.2809, respectively. The Runx2 and Osx expressions were significantly lower than those in the blank group (*P* <0.01). No difference was found in the Wnt expression (Table 3).

**Table 3:** **Gene expression in OB exposed to CCL3 and anti-CCL3**

	**Blank**	**CCL3**	**Anti-CCL3**
RUNX2	1.000 ± 0.0000	0.4002 ± 0.3734^*^	0.8670 ± 0.6827^#^
Wnt	1.000 ± 0.0000	0.7341 ± 0.4432	0.8246 ± 0.3579
Osterix	1.000 ± 0.0000	0.5242 ± 0.2809^*^	0.7993 ± 0.2468^#^

^*^Significantly lower than that in the blank group. ^#^Significantly higher than that in the CCL3 group.

### Partial restoration of OB function by CCL3 antibody

The effect of the anti-CCL3 was observed on the OB in vitro. The OCN level (787.358 ± 32.063 μg/L) and the amount of mineralized nodules (6.88 ± 1.75/HPF) were higher than those in the CCL3 group. However, no statistical significance was found (Table 2). The mRNA expressions of Runx2 and Osx after the anti-CCL3 antibody was added were 0.8670 ± 0.6827 and 0.7993 ± 0.2468, respectively. These expressions were significantly higher than those of the CCL3 group (*P* <0.05). By contrast, the Wnt mRNA expression had no significant difference under the same condition (Table 3). The anti-CCL3 antibody partly restored the OB differentiation by increasing the function of the transcription factors.

### Discussion

MM remained an incurable disease ultimately leading to the death of all patients despite the significant progress in understanding the MM biology and the promising advances in the development of treatment strategies. Osteolytic bone lesions still remained a therapeutic challenge. The histomorphometric studies conducted in the patients with MM with bone lesions showed that bone remodeling was severely imbalanced because the increased bone resorption was not counterbalanced by the deposition of new bone tissues [10]. The OB activity was affected by a series of mechanisms including the abnormal production of cytokines, chemokines, and inflammatory factors, which usually increased in the marrow microenvironment [11]. Abnormal OBs were also proven associated with the imbalanced cellular immunity in MM [12]. Moreover, deregulated osteoblastogenesis was promoted by the direct OB interactions with the myeloma cells. This interaction severely suppressed both proliferation and differentiation through the release of a number of inhibitory factors such as Dickkopf-1 [13] and parathyroid hormone-related protein [14].

The mechanism underlying the suppression of the OB activity was not explained well because of the difficulty of the human OB culture that limited the investigations. We cultured MM-derived OBs in vitro and provided a medium containing dexamethasone, vitamin C, and β-sodium glycerophosphate. Dexamethasone regulated gene expression in differentiating cells and induced the affinity of the glucocorticoid receptor for its target sequence in the genome [15]. The bone cells were the target of the glucocorticoid hormones. Vitamin C was required for the synthesis of collagen [16] and osteogenesis in vitro [17]. Anderson^19^ demonstrated that vitamin C regulated ATPase and ATP activities and protein synthesis in cultures of osteoblast-like cells. β-Sodium glycerophosphate was often used in vitro to provide a potential source of phosphate ions [18].

OB inhibition was frequent in the bone biopsies of patients with MM and was correlated with tumor burden [19]. We have demonstrated that the OBs, which were isolated from the BM in patients with MBD, showed a decrease in quantity and mineralized activity compared with the normal OBs of the healthy donors. The DT of the MM-derived OBs was longer than that of the normal controls. In agreement with the previous study [20], these results suggested that the quantity and the proliferative rate of the OBs from patients with MM were constitutively suppressed.

Recent studies have further reported that CCL3 (MIP-1α) was an osteoclastogenic C–C chemokine constitutively secreted by most of the MM cells in patients with multiple osteolytic lesions. The results of in vitro and in vivo studies showed that CCL3 induced OC formation in bone marrow cultures [21-23]. CCL3 acted as an OC-stimulating factor in the human BM cultures and was over-expressed in patients with MM but not in healthy individuals [24]. We further showed that CCL3 contributed to the OB/OC imbalance by promoting OC activity and by inhibiting OB activity.

In addition to OCs, OBs also expressed CCR1 receptors. The MM-derived OBs expressed higher CCR1 levels than those in the normal controls. Accordingly, CCL3 was involved in OB inhibition. We applied ELISA to the OCN levels detected in the supernatant of the MM-derived OBs exposed to CCL3 to verify whether CCL3 inhibited OBs. The analysis showed that CCL3 downregulated the OCN expression,which is a late and most cell-specific OB marker and also absent in endochondral and membranous skeletal elements [25]. The CCL3 exposure also impaired mineralization. The number of mineralized bone-like nodules of the OB in CCL3 was less than that of the blank controls, which suggested that the OB function was inhibited after CCL3 treatment. Vallet et al. [26] showed that mature OB from primary cells and human stromal cell line (i.e., HS27A) expressed higher levels of CCR1 and CCR5 mRNA than immature OB. They concluded that the receptor expression observed on the mature cells accounted for the preferential inhibition of OB function rather than differentiation. We analyzed the OCN concentration to assess the OB function and the mRNA expression of both Runx2 and Osx and to evaluate the degree of OB differentiation.

The OB maturation from mesenchymal cells was primarily regulated by Runx2 [27]. The downstream factor Osx [28] played a role in the late OB differentiation by promoting the transcription of downstream genes such as OCN, osteopontin, and bone morphogenetic proteins [29]. The gene expression analysis of Runx2 and Osx revealed a downregulation in the CCL3-treated OBs, which supported the important role of CCL3 in inhibiting OB differentiation and maturation. However, the exposure to CCL3 did not significantly alter the expression levels of Wnt. We acknowledged that canonical Wnt signaling governed the lineage commitment and differentiation of progenitor cells into chondrocytes and osteoblasts [30]. Accordingly, Tripti [31] suggested that canonical Wnt signaling promoted osteogenesis by directly stimulating the Runx2 gene expression, which is expressed earlier than the Runx2. The undifferentiated Wnt expression indicated that CCL3 did not affect the OB differentiation in the early developmental stages of bone formation. Future studies should confirm these effects by using a larger sample size.

We exposed MM-derived OBs to CCL3 in the presence of neutralizing CCL3 antibodies to confirm the relevance of this chemokine in the MM-induced OB impairment. The CCL3 antibody treatment partially restored the Runx2 and Osx expressions in the OBs of patients with MM. The OCN levels in the culture supernatant also increased. Accordingly, CCL3 played a significant role in suppressing OB differentiation and function.

## Conclusion

The OB inhibition induced by CCL3 is associated with the Runx2/Osx pathway and the suppression of mineralization activation and OCN expression. These findings are useful in developing OB promotion. Using the CCL3 inhibitor helps control bone destruction with OB/OC coupling in patients with MBD and improves the patients’ quality of life if OB inhibition is partly induced by CCL3.
